# Dermoscopic Examination of Generalized Lichen Nitidus in a Patient With Down Syndrome

**DOI:** 10.7759/cureus.104140

**Published:** 2026-02-23

**Authors:** Karina Okamura, Natsuko Aoki, Tomoko Uemura, Mayuko Yamamoto, Kozo Nakai

**Affiliations:** 1 Dermatology, Kochi University, Kochi, JPN; 2 Pediatric, Kochi University, Kochi, JPN

**Keywords:** dermatology, dermoscopy, down syndrome, lichen nitidus, pediatric

## Abstract

Down syndrome, also known as trisomy 21, is the most frequently identified chromosomal disorder. Patients with Down syndrome frequently present with a broad spectrum of dermatological conditions, including atopic dermatitis, nonspecific dermatitis, folliculitis, seborrheic dermatitis, and cutaneous infections. Several cases of generalized lichen nitidus in individuals with Down syndrome have been reported. However, dermoscopic findings have not been previously described in these cases. We report a case in which dermoscopy contributed significantly to the clinical diagnosis, illustrating its potential role in improving diagnostic accuracy.

## Introduction

Down syndrome, also known as trisomy 21, is the most frequently identified chromosomal disorder, occurring globally in approximately one out of every 1,000 live births [1]. Chromosome 21 contains roughly 200-300 genes, and the presence of an extra copy leads to increased gene expression, which may contribute to a wide range of associated medical complications [2,3,4]. Despite receiving relatively limited attention in research and clinical guidelines, dermatologic manifestations can be an important concern in individuals with Down syndrome [5,6]. In one study evaluating health conditions among young adults with Down syndrome, 56% of participants reported having skin-related findings [7], including atopic dermatitis, nonspecific dermatitis, folliculitis, seborrheic dermatitis, and cutaneous infections [8]. These skin manifestations are typically evaluated initially by pediatricians; however, the diagnostic frequencies reported by pediatricians often differ from those identified by dermatologists. This discrepancy suggests that certain dermatological diseases may be underrecognized or misclassified in pediatric practice due to subtle clinical features or limited use of specialized diagnostic tools. Among the conditions that may escape accurate recognition is lichen nitidus, an uncommon, chronic inflammatory dermatosis characterized by discrete, tiny papules. The lesions are usually skin-colored to slightly erythematous, shiny, and dome-shaped, typically measuring 1-2 mm in diameter. They most frequently involve the trunk, genitalia, and flexor aspects of the extremities, but may occasionally become generalized. Most patients are asymptomatic or experience only mild pruritus. Although the exact etiology remains unclear, the disease is generally benign and self-limited, often resolving spontaneously over months to years. Although several cases of generalized lichen nitidus in individuals with Down syndrome have been reported, dermoscopic findings have not been previously described. We report a case in which dermoscopy contributed significantly to the clinical diagnosis, illustrating its potential role in improving diagnostic accuracy.

## Case presentation

**Figure 1:**
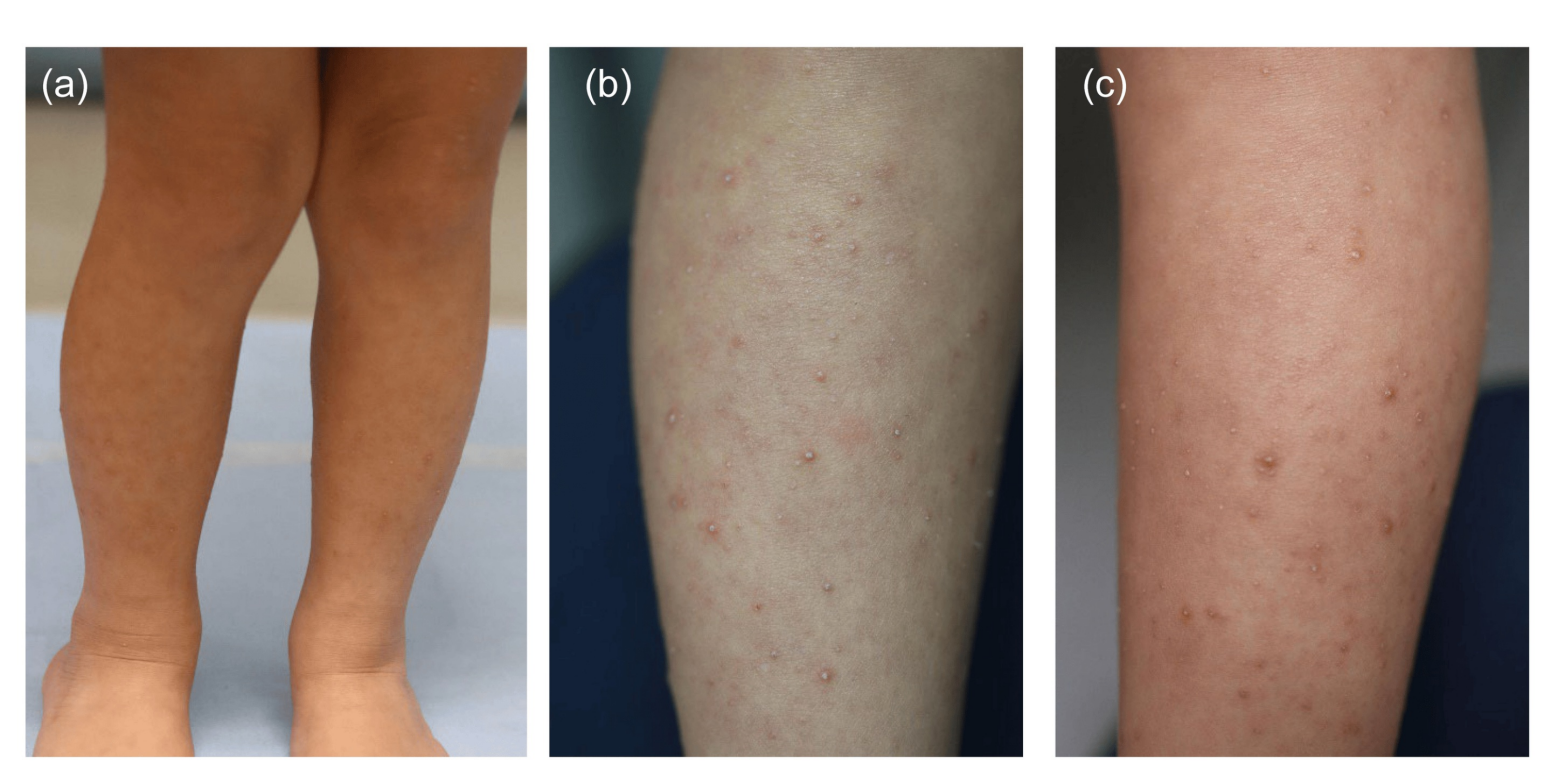
Clinical presentation of the patient. (a) Reddish-white, flat-topped, partially keratotic papules were observed on the limbs. (b) Magnified view of right leg. (c) Magnified view of left leg.

A six-year-old Japanese girl with Down syndrome was referred to our dermatology department with a persistent and progressively enlarging pruritic eruption that had been present for over one year. Prior to presentation, she had been treated with oral oxatomide and topical diflucortolone under presumed diagnoses of atopic dermatitis or chronic eczema; however, these therapies resulted in no clinical improvement. On examination, numerous (several hundred) reddish-white, flat-topped, partially keratotic papules measuring 1-2 mm in diameter were symmetrically distributed, predominantly on the flexor surfaces of the extremities (Figures 1a-1c).

**Figure 2:**
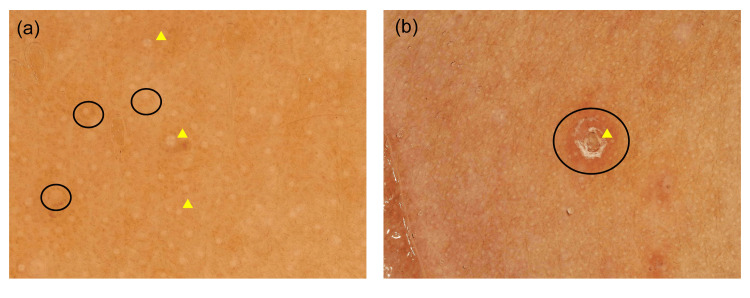
Dermoscopic examination (a) Numerous hypopigmentation (three representative lesions are indicated with arrowheads) accompanied by diffuse brown pigmentation (three representative lesions are encircled). (b) Papules with crusting (arrowhead) and erythema (encircled).

Dermoscopic evaluation revealed multiple, well-circumscribed, structureless hypopigmentation with brown shadow corresponding to the papules, surrounded by diffuse erythema and subtle red-brown pigmentation. No dotted vessels or scaling suggestive of eczema or folliculitis were observed. These findings are consistent with previously reported dermoscopic patterns of lichen nitidus, which represent focal inflammatory infiltrates under an acanthotic epidermis (Figure 2a-2b).

Although a skin biopsy was recommended to confirm the diagnosis histopathologically, her family declined the procedure. Based on the typical clinical morphology combined with dermoscopic characteristics, a diagnosis of generalized lichen nitidus was made. Continuing topical corticosteroids led to gradual improvement over several months, and no recurrence was observed during follow-up.

## Discussion

Patients with Down syndrome frequently present with a broad spectrum of dermatological conditions, including atopic dermatitis, nonspecific dermatitis, folliculitis, seborrheic dermatitis, and cutaneous infections. These cutaneous manifestations are often assessed initially by pediatricians; however, the diagnostic frequencies documented in pediatric practice may differ from those determined by dermatologists. This discrepancy implies that some dermatological disorders may be underrecognized or inaccurately categorized in pediatric settings because of subtle clinical presentations or limited application of specialized diagnostic tools. A recent multi-site retrospective study of 1,529 patients with Down syndrome identified eczematous dermatitis (26%) and folliculitis (19.3%) as the most prevalent dermatological conditions [8]. Although lichen nitidus is generally considered uncommon in the pediatric population, it has been repeatedly reported in association with Down syndrome. A systematic review estimated its prevalence in individuals with Down syndrome to be approximately 1.1%, suggesting that this condition may occur more frequently than in the general population [9]. According to a systematic review that included 40 observational studies and 99 case reports, lichen nitidus has been reported in approximately 1.1% of individuals with Down syndrome to date [10]. Because lichen nitidus can clinically mimic other papular dermatoses, such as follicular eczema or early eczematous eruptions, misdiagnosis may occur in the absence of dermoscopic or histopathological evaluation. Dermoscopy is regarded as a non-invasive and reliable method for evaluating lichen nitidus. According to a study of 20 cases with lichen nitidus, the dermoscopic findings were shiny elevated surface with absence of skin markings (100%), scaling (70%), radial ridges (50%) and central depression (35%), hypopigmentation (100%), brown shadow (70%), linear vessels (65%), accentuation of surrounding reticulate pigment network (65%), diffuse erythema (45%) [11,12]. Such features may be particularly useful when biopsy is impractical due to patient age, clinical circumstances, or caregiver preference. Although a biopsy was not performed in our case, the diagnosis was based on the characteristic clinical presentation and typical dermoscopic findings, which were consistent with previously reported features of lichen nitidus. When diagnosing lichen nitidus based on dermoscopic findings, several papular dermatoses should be considered in the differential diagnosis, including folliculitis, lichen planus, papular eczema, keratosis pilaris, and verruca plana (Table 1).

**Table 1:** Differential diagnosis of lichen nitidus

Disease	Dermoscopy
Folliculitis	Central follicular pustule or yellow-white structure.
Lichen planus	Wickham striae (whitish reticular lines) over a violaceous background with characteristic vascular patterns. The papules are usually larger and polygonal compared with lichen nitidus.
Papular eczema (lichenified dermatitis)	Dotted vessels and yellowish scales, usually accompanied by marked pruritus and less uniform papules.
Keratosis pilaris	Characterized by follicular keratotic plugs with perifollicular erythema. Coiled hairs and perifollicular scaling.
Verruca plana	Dotted or globular vessels on a light brown or yellowish background.

Importantly, while several cases of generalized lichen nitidus in Down syndrome have been documented, dermoscopic descriptions have not been previously reported, as shown in Table 2.

**Table 2:** Reported cases of lichen nitidus in patients with Down syndrome

	Journal	Year	Author	Sex	Age	Distribution	Dermoscopy	Biopsy
Case 1	Indian J Dermatol Venereol Leprol	2009	Agarwal et al.	Female	4	Legs, thigh	Not perfomed	Performed
Case 2	An Bras Dermatol	2012	Botelho et al.	Female	4	Face, trunk, upper and lower limbs, genitalia	Not perfomed	Performed
Case 3	SKINmed	2019	Guliani et al.	Male	Preschool	Whole body	Not perfomed	Performed
Case 4	Pediatr Dermatol	2009	Henry et al.	Male	3	Whole body	Not perfomed	Not perfomed
Case 5	J Eur Acad Dermatol Venereol	2006	Laxmisha et al.	Female	2	Whole body	Not perfomed	Performed
This case				Female	6	Upper and lower limbs	Performed	Not perfomed

Our case demonstrates that dermoscopy can provide valuable diagnostic support, especially when biopsy is impractical. This non-invasive strategy may improve diagnostic accuracy.

## Conclusions

We describe a case of generalized lichen nitidus in a child with Down syndrome diagnosed using dermoscopy in the absence of histological confirmation. This case highlights the importance of dermoscopic assessment in improving diagnostic precision and underscores its utility as a non-invasive method in clinical scenarios where biopsy cannot be performed. Increased awareness of this entity among pediatricians and dermatologists may facilitate earlier recognition, avoid unnecessary treatments, and contribute to a more accurate epidemiological understanding of dermatological manifestations in patients with Down syndrome.
